# Antibiotic resistances from slaughterhouse effluents and enhanced antimicrobial blue light technology for wastewater decontamionation

**DOI:** 10.1007/s11356-023-29972-x

**Published:** 2023-09-29

**Authors:** Xiaoyu Cong, Peter Krolla, Umer Zeb Khan, Mykhailo Savin, Thomas Schwartz

**Affiliations:** 1 Microbiology/Molecular Biology Department, Institute of Functional Interfaces (IFG), Karlsruhe Institute of Technology (KIT), Hermann von Helmholtz Platz 1, 76344 Eggenstein-Leopoldshafen, Germany; 2 Bioengineering Department, Faculty Life Sciences, Rhein-Waal University of Applied Sciences, Marie Curie Straße 1, 47533 Kleve, Germany; 3 Institute for Hygiene and Public Health (IHPH), Medical Faculty, University of Bonn, Venusberg-Campus 1, 53127 Bonn, Germany

**Keywords:** Antibiotic resistances, Wastewaters, Antimicrobial bluelight, Photo-sensitizer, Inactivation

## Abstract

The frequencies of 6 different facultative pathogenic bacteria of the ESKAPE group (priority list WHO) and a total of 14 antibiotic resistance genes (ARGs) with different priorities for human medicine were quantified in wastewaters of poultry and pig slaughterhouses using molecular biological approaches. Raw sewage from poultry and pig slaughterhouses was found to be contaminated not only with facultative pathogenic bacteria but also with various categories of clinically relevant ARGs, including ARGs against the reserve antibiotics group. The concentration of the different gene targets decreased after on-site conventional biological or advanced oxidative wastewater treatments, but was not eliminated. Hence, the antimicrobial BlueLight (aBL) in combination with a porphyrin photo-sensitizer was studied with ESKAPE bacteria and real slaughterhouse wastewaters. The applied broad LED-based blue light (420–480 nm) resulted in groups of sensitive, intermediate, and non-sensitive ESKAPE bacteria. The killing effect of aBL was increased in the non-sensitive bacteria *Klebsiella pneumoniae* and *Enterococcus faecium* due to the addition of porphyrins in concentrations of 10^−6^ M. Diluted slaughterhouse raw wastewater was treated with broad spectrum aBL and in combination with porphyrin. Here, the presence of the photo-sensitizer enhanced the aBL biocidal impact.

**Supplementary Information:**

The online version contains supplementary material available at 10.1007/s11356-023-29972-x.

## Introduction

Antimicrobial resistance (AMR) is one of the greatest health challenges of our time and has significant economic consequences for society (WHO 2017; 2019a; 2020). The WHO documented worldwide that about 700,000 deaths per year are caused by antibiotic resistant bacteria (ARB) (WHO 2019a). Furthermore, it is predicted that their number will increase to nearly 10 million in 2050 (de Kraker et al. 2016).

The global spread of AMR is mainly due to the emission of ARB, ARGs, facultative pathogenic bacteria, and AMR-causing substances in human and animal waste into the environment. In this context, it is also important to mention the selective pressure via antibiotic misuse and overuse, which is an important aspect for the evolution and spread of ARB. Recently, much effort has been devoted to deciphering the sources, transmission pathways, and sinks of AMR in a series of AMR screening studies around the world (Marano et al. 2020; Pärnänen et al. 2019; Cacace et al. 2019). Major hotspots of anthropogenic AMR include (i) point sources, e.g., hospitals, nursing homes, private households, pharmaceutical industries, animal husbandries, and slaughterhouses (Voigt et al. 2020; Savin et al. 2020); (ii) urban wastewater treatment plants (Alexander et al. 2020); and (iii) other diffuse sources (Alexander et al. 2020; Hembach et al. 2022; Paulus et al. 2019; Schwermer et al. 2018; Amos et al. 2014a; Amos et al. 2014b).

Wastewater from AMR hotspots is mostly discharged into the public sewerage system, which plays an important role as a recipient of potentially harmful and AMR-promoting substances as well as ARBs and ARGs (Alexander et al. 2022). Therefore, it acts not only as an incubator for the emergence of AMR but also as a pathway for the transfer of ARBs and ARGs from their sources to municipal wastewater treatment plants (WWTP). Currently, the global widespread use of antibiotics in animal husbandry has sped up the prevalence and occurrence of AMR and posed massive issues for global human health (Hembach et al. 2022; He et al. 2020). Over 80% of the meat consumption per person in Germany comprised pork and poultry. Furthermore, these two animal species receive nearly 80% of all antibiotics used in the livestock industry in Germany (Schwaiger et al. 2012; Schwarz and Chalsus-Dancla 2001; Hembach et al. 2022). The wastewater from slaughterhouses is believed to contain ARB and ARGs of clinical relevance that cannot be totally eliminated by means of conventional treatment techniques (Savin et al. 2020). As a result, different clinically relevant AMR may be present in municipal sewer systems and subsequently in the effluent of WWTPs reaching the aquatic environment. Through horizontal gene transfer, ARB and ARGs can spread swiftly throughout the environment indigenous bacteria, which have a detrimental effect on human health in case of transfer to hygienically relevant bacteria. It is unknown whether resistance-causing bacteria enter sewer systems more frequently at the point of slaughter compared to municipal or clinical emitters.

Different clusters occurring in different (commonly, intermediately, and rarely) abundacies of ARGs were previously identified not only in wastewater but also in anthropogenically influenced surface waters (Hembach et al. 2019). Here, the commonly occurring resistance gene clusters with *erm*B, *tet*M, *bla*_TEM_, and *sul*1 directed against macrolids, tetracylines, β-lactams, and sulfonamide antibiotics are listed. The cluster with the intermediately occurring resistance genes *bla*_CTX-M_, *bla*_CTX-M-32_, *bla*_OXA-48_, and *bla*_CMY-2_ coding for cephalosporine (2nd generation), and carbapeneme antibiotic resistances. The most critical cluster contains rarely occurring resistance genes against reserve antibiotics used in human therapies (*bla*_VIM_, *van*A, *mec*A, *mcr*-1, *bla*_NDM_), i.e., against carbapenems (e.g., imipenem), gycopeptides (e.g., vancomycin), methicilline (MRSA), and cyclic peptides (e.g., colistin) (Hembach et al. 2019; Alexander et al. 2020).

The effect of blue light as an antimicrobial active driver, referred to as “antimicrobial blue light-aBL,” has been known for some time, but remained largely unexploited (Leanse et al. 2022; Wang et al. 2017). Most of the studies are linked with hospital settings for the decontamination of wounds or other biotic and abiotic surfaces (Wainwright 1998; Hamblin and Hassan 2004). In addition, antimicrobial blue light treatment is also used in the food industry and in multiple barrier technologies in conditioning processes (Hadi et al. 2020). The blue light was shown in many studies to inactivate a wide range of pathogenic bacteria, regardless of their antibiotic resistance profile, Gram behavior, or other physiological specificities (MacLean et al. 2009). Inactivation has been demonstrated on biofilms in addition to planktonic life forms (Ferrer-Espada et al. 2019; Ferrer-Espada et al. 2020). Based on these facts, the use of a broad blue spectrum emitting light source has been selected to excite the different photo-sensitive structures, leading to a non-specific and sustained killing effect on bacterial cells, especially of the ESKAPE group (Hoenes et al. 2019, 2021). Different targets in bacterial cells like DNA, proteins, lipids, and the cell membranes are impacted by aBL (Dos Anjos et al. 2023). So far, less is known about results of an irradiation of a broad aBL spectrum to bacterial cells and an evaluation of inactivation effects especially in industrial wastewater systems. In current publications, only light of a certain wavelength or a narrow wavelength range, mainly emitted from LED light sources, is used as aBL for irradiation (Maclean et al. 2009). Frequently, aBL with emission wavelengths around 405 nm or 415 nm is used to excite endogenous porphyrin-containing molecules. Wavelengths around 450 nm are also known to excite the cytochromes of the P450 complex. These cytochromes consist of porphyrin-containing molecular clusters, mostly photo-sensitive porphyrinogenic precursors. P450 classes catalyze the terminal reduction of oxygen of the electron transfer chain (ETC) as oxidoreductases. Their function can be disturbed by light in the 450 nm range, leading to the death of bacterial organisms. In addition, wavelengths of 410–435 nm are still used as antibacterially effective, based on flavin-containing proteins and molecules of different oxidation states (Plavskii et al. 2018).

Blue light with co-applied exogenously administrations of photo-sensitizer such as prophyrin derivatives are already in use in medical photo-dynamic therapies (PDT). Such systems are used clinically in the field of wound healing and cancer therapy, where they also show great success in combating bacterial infections (Leanse et al. 2022; Wang et al. 2017; MacLean et al. 2008). Beside the antibacterial impact, effective LED-based broad-spectrum antiviral treatments in combination with light-stimulated photo-sensitizers were demonstrated by Heffron et al. (2021). The reason for the antibacterial effect of blue light is its interaction with endogenous, photo-sensitive molecules (heme proteins, cytochromes, flavoproteins, oxygenases, etc.). These photo-sensitive molecules react under light absorption with an energy or electron transfer to other molecules in the direct vicinity. The formation of the so-called reactive oxygen species (ROS), such as singlet oxygen (O_2_^1^), superoxide anions (O_2_^−^), and the highly toxic hydroxyl anion (HO^−^) affect negatively vital structures of bacterial cells (i.e., membranes, protein structures of the electron transfer chain (ETC), or antioxidative protective systems (Hamblin et al. 2004; Wainwright et al. 1998).

It is believed that the wastewaters from slaughterhouses contain bacteria and genes with the ability to spread zoonotic and human health relevant diseases (Savin et al. 2020). To prevent and decrease serious health issues caused by the dissemination of ARB and ARGs, the current investigations examined the prevalences of ESKAPE-group bacteria, various classes of clinically significant ARGs, and how exposure to both conventional treatment methods and ozone affected the abundance of each gene target. Therefore, one objective of this study is the determination of the occurrence and abundances of 21 gene targets in the wastewater samples collected from poultry and pig slaughterhouses on various sampling days and sampling locations. We are focusing on the occurrence of Gram-negative and Gram-positive members of the ESKAPE bacteria (WHO 2017). ESKAPE is an acronym comprising the scientific names of six highly virulent and antibiotic resistant bacterial facultative pathogens including *Enterococcus faecium*, *Staphylococcus aureus*, *Klebsiella pneumoniae*, *Acinetobacter baumannii*, *Pseudomonas aeruginosa*, and *Enterobacter spp.* Due to their heightened resistance to frequently used antibiotics, these facultative pathogenic bacteria pose an additional threat to the safety of the general population (Mulani et al*.* 2019). The second objective of this study is the impact of broad-spectrum LED blue light on these facultative pathogenic bacteria of the ESKAPE group. Here, the sensitivity of these bacteria is studied with and without photo-sensitizer application. The impact of high- and low-nutrient media on the aBl inactivation especially on bacteria from different growth phases is analyzed. Especially, blue light–insensitive reference bacteria and native slaughterhouse wastewater samples were treated with aBL together with porphyrin-based photo-sensitizer molecules for an inactivation of health critical bacteria.

## Material and methods

### Sampling and sample preparation

The wastewater samples were collected twice from a German poultry slaughterhouse on different days in summer time. Six samples were collected for each monitoring date: two samples from the raw wastewater that is the influent of an integrated and conventional wastewater treatment plant (WWTP), two samples from the effluent that is released from the conventional WWTP, and two samples from the effluent that has subsequently undergone an ozone treatment (75 g ozone/m^3^). The daily wastewater volume of the poultry slaughterhouse was about 3,600 m^3^. The average slaughter rate is > 100,000 chicken per day.

In case of a German pig slaughterhouse, the sample acquisition was conducted in two independent campaigns. Four samples were taken for each monitoring date: two from the raw wastewater that is the influent of an integrated physico-chemical treatment and 2 samples from the effluent that is released from the physico-chemical facility containing a floatation and precipitation treatment of particulate matter. The slaughterhouse exhibits a slaughtering capacity > 10,000 pigs per day. Daily wastewater volume of about 2,100 m^3^ is treated by a physical-chemical and biological WWTP using flotation and precipitation (flocculation).

After these on-site pre-treatments, the conditioned wastewater of pig slaughterhouse is released into a municipal WWTP via public sewer systems and the wastewater from the poultry slaughterhouse is discharged into a river receiving body.

The samples from poultry and pig slaughterhouses were transported to the laboratory via Express service at 4–7 °C for max. 24 h before being further processed. Following that, vacuum filtration was used to separate the wastewater liquids from suspended cellular matter including bacteria. Polycarbonate membranes (Ø 47 mm, pore size 0.2 μm, Whatman Nucleopore Track-Etched Membranes, Sigma-Aldrich, Munich, Germany) were used together with a sterilized filtration unit. Since the filtration volume depends on the turbidity, the volumes of each sample were 100 mL after conventional treatment, 200 mL for the effluent (poultry) after ozonation, 100 mL for the effluent (pig) after biological treatment, and 30 mL for the raw wastewater being the influent from WWTPs (i.e., raw wastewater).

### Live/dead discrimination using propidium monoazide (PMA)

Propidium monoazide (PMA) treatment can be utilized to distinguish intact bacteria from dead or injured bacteria (Jäger et al. 2018). Damaged bacteria membranes are penetrated by PMA, where it intercalates with internal cellular DNA and is irreversibly crosslinked by a photo-activation step with light. This crosslink reaction effectively inhibits any subsequent PCR amplification (Nocker et al. 2007a, 2007b). After vacuum filtration, the filter membranes were treated with 25 μM PMA solution (Biotium, Haywards, CA, USA). The polycarbonate membranes were submerged in 2-mL colorless tubes (SafeSeal tubes, Carl Roth, Karlsruhe, Germany) containing the mixture of PMA and DNA-free water. Following a 5-min incubation period in the dark, PMA-treated samples were subjected to the PhAST Blue Photo-Activation System (GenIUL, Barcelona, Spain) at 100% intensity for 15 min to enhance the PMA crosslinking with DNA.

### DNA extraction for molecular biology analysis

DNA was extracted by using the FastDNA^TM^ Spin Kit for Soil (MP Biomedicals, Santa Ana, USA) and FASTPREP® instrument (MP Biomedicals, Santa Ana, USA). For mechanical cell disruption, the filtrated membranes were put in the Lysing Matrix E tube. Proteins were then separated by centrifugation and precipitation, and the DNA was finally purified by attaching to a silica matrix. The concentration of the extracted DNA was measured by NanoDrop (ND-1000, PEQLAB Biotechnologie GmbH, Germany) and the Quant-iT^TM^ PicoGreen® dsDNA Assay Kit (Thermo Fisher Scientific, Nidderau, Germany).

### Quantitative PCR analysis

SYBR Green qPCR tests were performed using a Bio-Rad Cycle CFX96 (CFX96 TouchTM Deep Well Real-Time PCR Detection System, Bio-Rad, Munich, Germany), and the analysis was done using the manufacturer’s software (Bio-Rad CFX Manager Software). Reactions were run in volumes of 20 μL, containing 10 μL Maxima SYBR Green/ROX qPCR Master Mix (2×) (Thermo Scientific Nidderau, Germany), 7.4 μL nuclease-free water (Ambion, Life technologies, Karlsbad, Germany), 0.8 μL of Primer FW (10 μm), 0.8 μL of Primer Rev. (10 μm), and 1 μL of template DNA. The denaturation phase converts double-stranded DNA into single strands by heating up to a high temperature (about 95 °C) for 10 min. This was followed by 40 cycles of 15 s at 95 °C and 60 s at 60 °C. Melting curves were recorded by raising the temperature from 60 to 95 °C (1 °C every 10 s) to assess the specificity of the application. For each target either facultative pathogenic bacteria or specific antibiotic resistance gene, the primer sequences used are listed in SI Table 1. Information about the characteristics and quality of the qPCR systems used for the taxonomic genes as well as antibiotic resistance genes is given in SI Table 1. For the quantitative evaluation of an unknown sample, the cycle threshold (Ct value) measured in the qPCR is assigned to the number of cell equivalents with the help of the previously generated calibration line using reference bacteria. The number of cell equivalents of the dilution series is determined on the basis of the researched genome sizes and the measured DNA concentrations using the URL Genomics & Sequencing Center tool (http://cels.uri.edu/gsc/cndna.html). Different gene clusters for antibiotic resistances categorize the analyzed ARGs in frequently, intermediate, and rarely abundant gene targets and based on previous experiences published in Hembach et al. (2017, 2022) and Alexander et al. (2020, 2022). The clinical relevance of the investigated ARGs and facultative pathogenic bacteria and their percentage share in clinical infections is given in Table 1. All qPCR data are listed in SI Table 2 and SI Table 3 in Supporting Information section.

**Table 1:** Clinical relevance of the investigated antibiotic resistance genes and facultative pathogenic bacteria with their percentage share in clinical infections in 2016 according to data from the National Reference Centre for Surveillance of Nosocomial Infections (Aghdassi et al. 2016)

Antibiotic resistance genes	Antibiotic classes concerned
*erm*B	Macrolide antibiotic, erythromycin
*int*I1	Integron type 1 mobile genetic element (MGE)
*tet*M	Tetracycline antibiotic
*bla*_TEM_	Penicilline antibiotic
*sul*1	Sulfonamide antibiotic
*bla*_CTX-M_	Cephalosporin of 2. generation
*bla*_CTX-M-32_	Cephalosporin of 2. generation
*bla*_Oxa-48_	Carbapeneme β-lactame antibiotics
*bla*_CMY-2_	Cephalosporin of 2. generation
*bla*_VIM_	Carbapeneme, imipenem (reserve antibiotic)
*van*A	Glykopeptide, vancomycin (reserve antibiotic)
*mec*A	Penicilline, methicillin resistance (multi-resistance in *S. aureus*)
*mcr*-1	Cyclic peptide antibiotic, colistin (reserve antibiotic)
*bla*_NDM_	Resistance against multiple β-lactame antibiotics, carbapeneme
Facultative pathogenic bacteria	Clinical relevance
*Enterococci* spp. (23S rDNA)	14.3% of clinical infections
*Enterococcus. faecalis (dll* gene)	6.9% of clinical infections
*Pseudomonas aeruginosa* (*ecfX* gene)	5.8% of clinical infections
*Klebsiella pneumoniae* (*glt*A gene)	4.5% of clinical infections
*Acinetobacter baumannii* (*sec*E gene)	5% of clinical infections
*Escherichia coli* (*ycc*T gene)	16.6% of clinical infections

### Cultivation of ESKAPE facultative pathogenic bacteria for aBL irradiation

Bacterial strains were initially cultivated overnight in 40 mL high nutrient medium LB-medium (Luria-Bertani, Sigma-Aldrich, Darmstadt, Germany) at 37 °C (stationary phase). In a second approach, freshly grown on the day of use bacterial suspensions from the exponential growth phase were used for aBL irradiation. Bacteria were washed three times with PBS solution (phosphate buffered saline) including centrifugation and resuspension steps. The washed bacterial pellets were resuspended in 20 mL PBS solution to an optical density of about OD_(600 nm)_: 0.1 (Hitachi Photometer, Hitachi-High-Tech Cooperation, Tokyo, Japan) which corresponds to about 5.0 × 10^8^ colony forming units (CFU)/mL. Alternatively, low nutrient mineral medium BM2 (i.e., 100 mL 10× BM2, 10 mL glucose (40 % w/v), 10 mL MgSO_4_ (200 mM), 1 mL FeSO_4_ (10 mM) in 1 L sterile dH_2_O; freshly prepared before use) was used to study the bacteria inactivation during aBL application. Both bacterial suspensions from stationary as well as exponential growth phases were treated and analyzed.

### Experimental setup for aBL and photo-sensitizer treatment

For aBL illumination experiments, sterile 20-mL glass vials together with a sterilized magnetic stirrer bar (Ø 2 mm * 5 mm) were used for a 5 mL volume approach. Therefore, 0.5 mL of the freshly washed bacterial suspension of OD_(600 nm)_ ~ 0.1 was pipetted to 4.5 mL PBS solution, resulting in a 10-fold dilution step with a final CFU of about 5.0 × 10^7^ per mL. Dark control experiments were performed in parallel by covering the vials tightly with a foil.

For the illumination of the bacterial suspensions, 4 conventional 8 W LED bars (SolarStinger, SunStrip, DeepBlue, Econlux, Germany) were used. Equipped with a repeating sequence of 4 different LED types (SI Fig. 1) emitting wavelengths of 400 nm, 420 nm, 440 nm, and 460 nm, these LED bars were emitting an entire spectrum of blue light (SI Fig. 1). Due to the predetermined light source composition, a modulation of specific aBL emitting LED types was not possible. Sample vials were center placed on a multi-position magnetic stirrer (2Mag MixDrive60; Munich, Germany) and 1000 rpm was adjusted. The vials were located between two light source units in a refrigerated incubator at 30 °C for the entire exposure time (Thermo MaxQ 4000; Thermo-Fischer Scientific, Darmstadt, Germany). As temperature control, a separate vial containing liquid medium linked with a temperature sensor was run in parallel. The refrigerator setup helped to avoid any temperature-mediated impact on bacteria during blue light exposition. Radiant intensity data were collected with a calibrated Spectrophotometer (FLAME-S-XR1-ES, with optical fiber QP400-2-SR-BX; OceanInsight, Ostfildern, Germany) for the LED light source inside the incubator. As measurements inside a reaction vials in liquid could not be performed, the radiant intensity was measured at the vial position in air. Due to the fixed light emission of the LEDs, different light intensities, high and low, were performed by changing the light source and reaction vial distance. The conversion of the radiant intensity in W/cm^2^ used into energy intensity in J/s *cm^2^ is given for the applied high and low intensities in SI Table 4. Here you will also find the energy densities calculated for the time-dependent irradiations (1–4 h).

Irradiation measurement values at 245 and 543 W/m^2^ were chosen for aBL irradiation experiments. The photo-sensitizer 5, 10, 15, 20-Tetrakis-(N-Methyl-4-pyridyl) 21, 23-porphyrin tetratosylat (TMPyP; CAS: 36951-72-1) was purchased from Sigma-Aldrich (Darmstadt, Germany). A 1 × 10^−3^ M stock suspension of TMPyP photo-sensitizer was prepared with PBS and stored at 4 °C. The base structure of the planar organized, poly-cationic TMPyP molecule is a porphyrin ring. Porphyrin molecules adsorb light at a specific spectrum. The so-called Soret-band indicates the wavelength range of maximum adsorption, which is about 422 nm for TMPyP suspended in PBS. The TMPyP photo-sensitizer was premixed together with the bacterial test suspensions, followed by a 30 min preincubation step in dark before aBL irradiation.

A defined aliquot was taken out of the treated bacterial suspensions. Subsequently up to six ten-fold dilution steps were performed. From each dilution sample, 5 × 10 μL aliquots (droplets) were pipetted onto LB plates. The incubation time was 24 h and the temperature was adjusted to 37 °C depending on the used reference bacteria. After incubation the numbers of colonies grown on the agar plates were counted and statistically analyzed to colony forming units (CFU) per mL.

### Slaughterhouse raw wastewater treated with aBL and porphyrin photo-sensitizer

A total of four raw wastewater (RWW) samples from a poultry slaughterhouse were studied. The RWW samples were directly collected from the influent to the slaughterhouse on-site wastewater treatment plant and had not undergone any chemical or conventional treatment.

The RWW were centrifuged at 1500 rpm for 1 min (Eppendorf 5810R refrigerated centrifuge) to sediment the rough particles. The optical densities (OD) of the samples were then determined at 600 nm using an spectro-photometer (Hitachi U-5100). The OD_600_ values of the undiluted samples were about 1.8 for the chicken and 1.9 for the pig slaughterhouse samples. Therefore, the samples were then diluted 1:10 in PBS (phosphate buffer saline, pH 7.4). The OD values were about 0.3 after dilution. The diluted samples were added to 5-mL glass vials with a 5-mm magnet and sealed with a suitable glass lid. The glass vials were then placed on a magnetic stirrer (25 Mag Emotion, Mixdrive 60) in an incubator at a temperature of 37 °C (Thermo MaxQ 4000). The glass vials with the diluted raw water samples were placed between 4 blue light bars on a magnetic stirrer. The magnet inside the glass vial rotated continuously to ensure an continous movement of liquid in the glass vials and prevent a sedimentation of the bacterial cells. The samples were treated with blue light up to 4 h (for dose calculation see SI Table 4).

## Results

### Abundances of facultative pathogenic bacteria and ARGs in wastewaters from poultry slaughterhouse

**Fig. 1:**
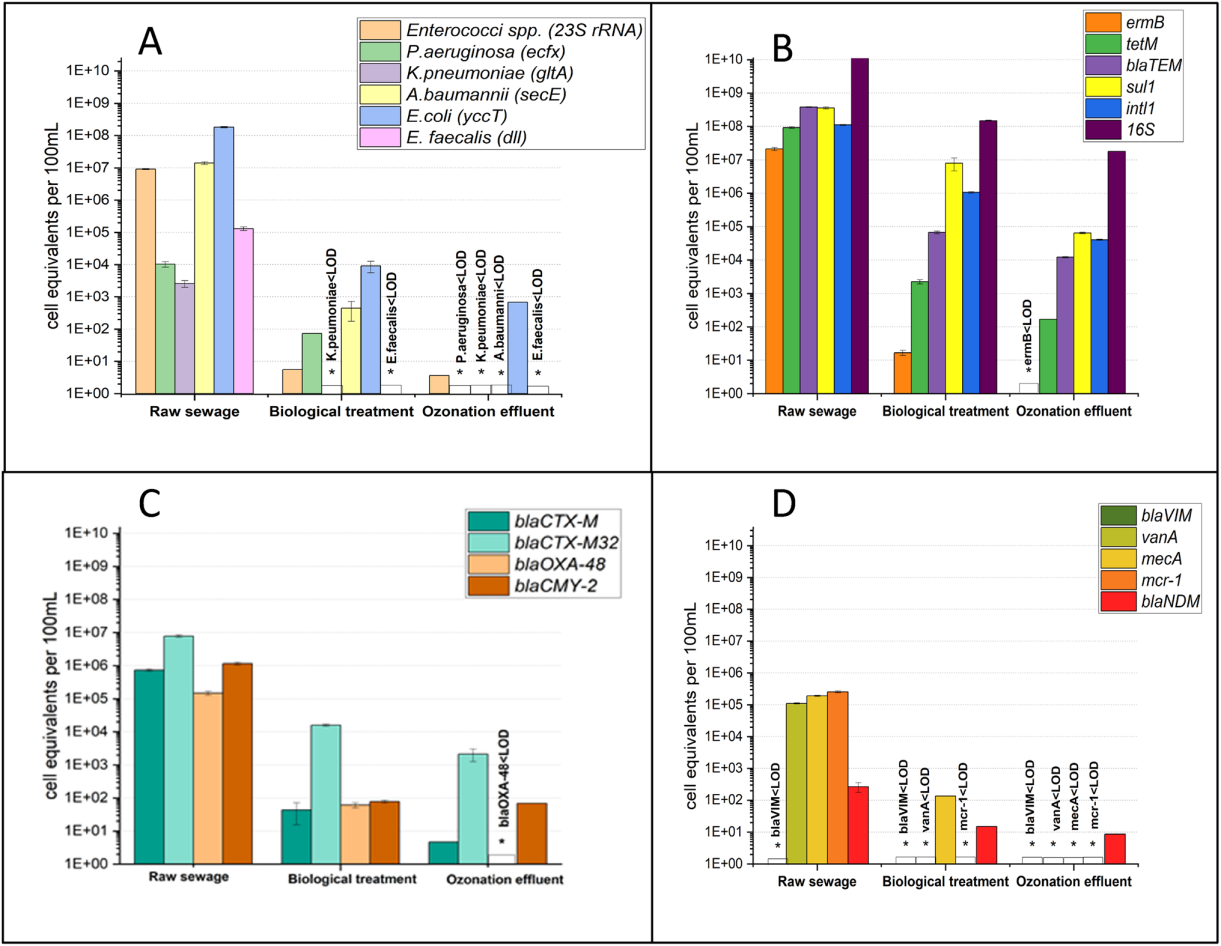
Water sampling from poultry slaughterhouse. Abundance of facultative pathogenic bacteria (**A**), “commonly” (**B**), “intermediately” (**C**), and “rarely” (**D**) occurring resistance genes. Gene targets for bacteria and ARGs were quantified by qPCR in raw wastewaters before and after on-site biological conventional wastewater treatments and ozone treatment. LOD, below limit of detection. The data from each sampling campaign including standards deviation are listed in SI Table 2

Following PMA treatment of the samples, the ESKAPE group bacteria were measured using species-specific gene markers and the results are presented in Fig. 1A. The most prevalent target species in the raw wastewater were *Escherichia coli*, followed by *Enterococcus spp*. and *A. baumannii*, with abundances of 1.8 × 10^8^, 9.2 × 10^6^, and 1.4 × 10^7^ gene copies/100 mL, respectively. The lowest concentration with 2.6 × 10^3^ gene copies/100 mL was quantified for *K. pneumoniae*. The abundances of *P. aeruginosa* and *E. faecalis* were 1 × 10^4^ and 1.3 × 10^5^ gene copies/100 mL, respectively.

*K. pneumoniae* and *E. faecalis* were reduced below the limit of detection (LOD) after the biological sludge treatment and subsequent ozonation, while *Enterococcus* spp., *P. aeruginosa, A. baumannii,* and *E. coli* were reduced in several log_10_ units by 6.5, 2.1, 4.2, and 4.2, respectively. Following the subsequent ozonation, *E. coli* and *Enterococcus spp.* demonstrated a lower decrease, whereas *P. aeruginosa* and *A. baumannii* exhibited a significant decrease below the LOD.

With the exception of the gene *bla*_VIM_, almost all ARG targets were present in the raw water coming directly from the poultry slaughterhouse. In the raw sewage (i.e., influent of the on-site WWTP), among all “commonly occurring resistance genes” (Fig. 1(B)), *bla*_TEM_ and *sul*1 exhibited the highest abundance of 3.8 × 10^8^ and 3.6 × 10^8^ gene copies/100 mL, respectively, followed by Integron-specific gene *intl*1 (1.1 × 10^8^) being involved in horizontal gene transfer, *tet*M (9.1 × 10^7^), and *erm*B (2.1 × 10^7^). All regularly occurring ARGs decreased by several log units after using conventional biological treatment technologies; the most notable decline was 6 log units in case of *erm*B. The ARGs *tet*M and *bla*_TEM_ were reduced by 4.6 and 3.7 log_10_ units, respectively. Similarly, *sul*1 and *Intl*1 gene targets were removed by 2 log_10_ units. The subsequent ozonation reduced the *erm*B gene target below the detection limit. The *sul*1 gene was reduced up to 2 log_10_ units, followed by *tet*M (1 log_10_ unit), *intl*1 (1.3 log_10_ units), and *bla*_TEM_ (0.7 log_10_ unit).

The abundance of intermediate abundant ARGs in the analyzed samples is shown in Fig. 1C. In raw water samples, *bla*_OXA-48_ (1.4 × 10^5^ gene copies/100 mL), *bla*_CMY--2_ (1.1 × 10^6^ gene copies/100 mL), *bla*_CTX-M32_ (7.8 × 10^6^ gene copies/100 mL), and *bla*_CTX-M_ (7.3 × 10^5^ gene copies/100 mL) were present in relatively high concentrations.

Regarding the samples from the biological treatment on site, each gene concentration decreased to varying degrees. The *bla*_CMY-2_ gene concentration was found with the strong reduction, with 4.1 log_10_ units, followed by *bla*_CTX-M_ (3.6 log_10_ unit), *bla*_OXA-48_ (3.3 log_10_ unit), and *bla*_CTX-M32_ (3 log_10_ unit). After ozonation of the previous biologically treated wastewater on site eliminated the *bla*_OXA-48_ gene below the LOD, whereas for the *bla*_CMY-2_ gene no significant reduction was observed. In addition, *bla*_CTX-M_ and *bla*_CTX-M32_ were both decreased by 1.2 log_10_ and 1 log_10_ units after ozonation, respectively.

Most of the rarely occurring clinically relevant ARGs were not detected in pre-treated poultry wastewaters after ozonation (Fig. 1D). The ARGs *mcr*-1, *mec*A, *van*A, and *bla*_NDM_ were detected in raw wastewater samples released by poultry slaughterhouse with comparable concentrations of 2.5 × 10^5^, 1.9 × 10^5^, 1.1 × 10^5^, and 2.6 × 10^5^ gene copies/100 mL. These results indicated the presence of clinically important ARGs released by the raw wastewaters. The conventional biological treatment technologies decreased gene concentrations of *bla*_NDM_ and *mec*A by 1.5 and 2.3 log_10_ units, respectively, and other targets were measured below the detection limits. Most of the ARG targets of this category were found to be below the detection limits after ozone treatment. Only *bla*_NDM_ that was still detectable, but was further reduced by 0.2 log_10_ units after ozonation.

Notably, high concentration of facultative pathogenic bacteria and different categories of ARGs are released via wastewater from poultry slaughterhouses. Despite the fact that these cell equivalent values decreased during on-site biological treatment and subsequent ozonation, the dissemination of hygienically relevant bacteria and ARGs continues to be serious issues and contribute to the spread of AMB. This applies especially to slaughterhouses that do not have effective wastewater treatment facilities. Here, insufficiently treated wastewater is discharged directly into the receiving waters or pollutes the municipal sewage treatment plants.

### Abundances of facultative pathogenic bacteria and ARGs in wastewaters from pig slaughterhouse

**Fig. 2:**
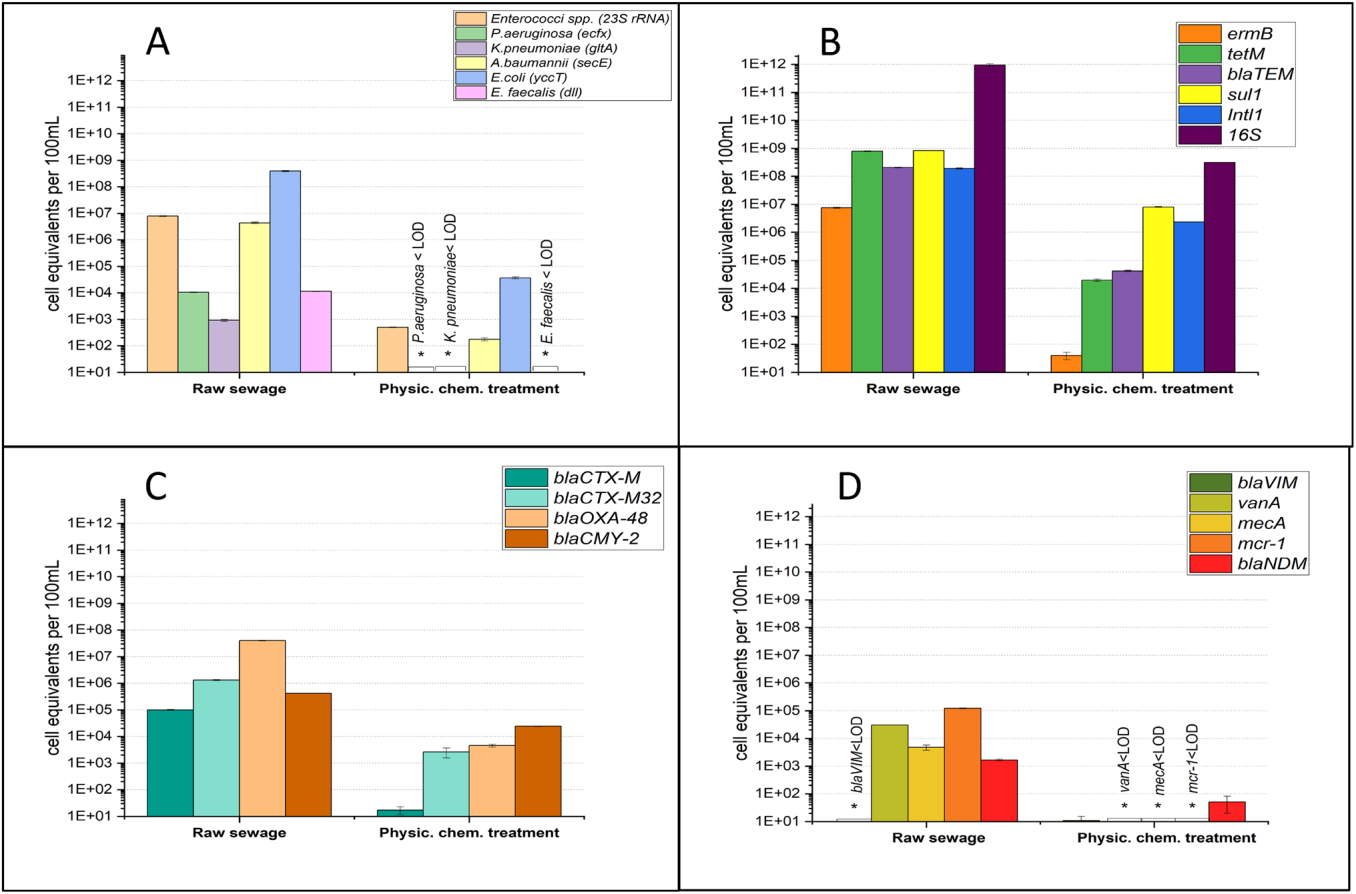
Water sampling from pig slaughterhouse. Abundance of facultative pathogenic bacteria (**A**), “commonly” (**B**), “intermediately” (**C**), and “rarely” (**D**) occurring resistance genes. Gene targets for bacteria and antibiotic resistance genes were quantified by qPCR in raw wastewaters before and after on-site physical chemical treatment. LOD, below limit of detection. The data from each sampling campaign are listed in SI Table 3

As seen in Fig. 2(A), *E. coli* (3.8 × 10^8^ gene copies per 100 mL) was the most abundant facultative pathogenic bacterium, followed by *Enterococcus* spp. (7.8 × 10^6^ gene copies/100 mL), *A. baumannii* (4.3 × 10^6^ gene copies/100 mL). *P. aeruginosa,* and *E. faecalis* had roughly equal relative abundances with 1.0 × 10^4^ and 1.1 × 10^4^ gene copies/100 mL, respectively. *K. pneumoniae* had the lowest concentration, measuring 9.3 × 10^2^ gene copies/100 mL. *P. aeruginosa, K. pneumoniae*, and *E. faecalis* were reduced after advanced treatment below the limit of detection (LOD), whereas *Enterococcus* spp., *A. baumannii*, and *E. coli* were all reduced in several log_10_ units by about 4.

Regarding the different clusters of ARGs (Fig. 2B, C, and D)*, tet*M (8 × 10^8^ gene copies/100 mL) and *sul*1 (8.3 × 10^8^ gene copies/100 mL) genes demonstrated high concentrations among commonly occurring resistance genes. The relative abundances of *bla*_TEM_ and integron *Intl*1 were approximately the same with 2.1 × 10^8^ and 1.9 × 10^8^ gene copies/100 mL, respectively. The gene *ermB* exhibited the lowest gene copy value in the raw wastewater, but showed the greatest reduction by 5.5 log_10_ units after biological treatment. Additionally, after being exposed to biological treatment, the eubacterial 16S rRNA gene marker was diminished by 3.5 log_10_ units from its initial concentration of 9.3 × 10^11^ gene copies per 100 mL. The ARGs *tet*M, *bla*_*T*EM_, *sul*1, and *Intl*1 also showed reductions of 4.5, 3, 2, and 1.5 log_10_ units, respectively.

Among intermediate occurring ARGs (Fig. 2C) consist of *bla*_CTX-M_ (9.9 × 10^4^ gene copies/100 mL), *bla*_CTX-M32_ (1.3 × 10^6^ gene copies/100 mL), *bla*_OXA-48_ (4 × 10^7^ gene copies/100 mL) and *bla*_CMY-2_ (4.1 × 10^5^ gene copies/100 mL). The ARG *bla*_CTX-M_ was found to be the most affected ARG by the biological treatment in this group due to the largest drop in its cell equivalent value, which is by 3.8 log_10_ units, followed by *bla*_OXA-48_ (3.1 log_10_ units), *bla*_CTX-M32_ (2.5 log_10_ units), and *bla*_CMY-2_ (1 log_10_ unit).

The final category consists of ARGs that were thought to be rare or to have only been detected in trace amounts in raw wastewaters or WWTP influent (Alexander et al. 2020; Hembach et al. 2017). Since this subset of ARGs was directed against last-resort antibiotics, we thought that these ARGs were the most critical among the targets being evaluated. These ARGs include *bla*VIM (lower than detection limit), *van*A (3 × 10^4^ gene copies/100 mL), *mec*A (4 × 10^3^ gene copies/100 mL), *mcr*-1 (1.2 × 10^5^ gene copies/100 mL), and *bla*_NDM_ (1.6 × 10^3^ gene copies/100 mL), which fall into the category of infrequently occurring resistance genes. Nevertheless, these gene targets together with their carrying bacteria can reach the aquatic environment in case of proper removal treatments at slaughterhouses. Hence, the dissemination of these resistance genes could become a serious public and environmental threat, when people are colonized with these ARG carriers. In fact, the concentration of the colistin resistance gene *mcr*-1 was up to 1.2 × 10^5^ gene copies/100 mL, indicating that the raw water samples were strongly contaminated as mentioned by other studies (Hembach et al. 2017; Savin et al. 2020; Alexander et al. 2020). The majority of the ARG targets (*van*A, *mec*A, and *mcr*-1) in this category were found to be below detection limits after biological treatment, and the cell equivalent number of the gene *bla*_NDM_ was decreased by 1.5 log_10_ units. Hence, the dissemination of these particularly critical resistance genes against reserve antibiotics (e.g., vancomycin, colistin, imipenem) is inhibited or completely reduced.

As mentioned before, it became obvious that raw sewage from poultry and pig slaughterhouses are highly contaminated not only with facultative pathogenic bacteria of the ESKAPE-group but also with different clusters of clinically relevant ARGs. It is obvious that all gene targets became less concentrated after physicochemical and biological treatments, and their abundances even significantly decreased after subsequent ozonation in case of the poultry slaughterhouse wastewater treatment. Hence, the implementation of treatment processes for an effective reduction of hygienically relevant bacteria and clinically important ARGs is recommended for slaughterhouses. The presented results did also show that combinatory technologies like biological treatment followed by ozonation are most successful aiming on a strong reduction of these microbiological parameters.

### Inactivation of ESKAPE-group reference bacteria

**Fig. 3:**
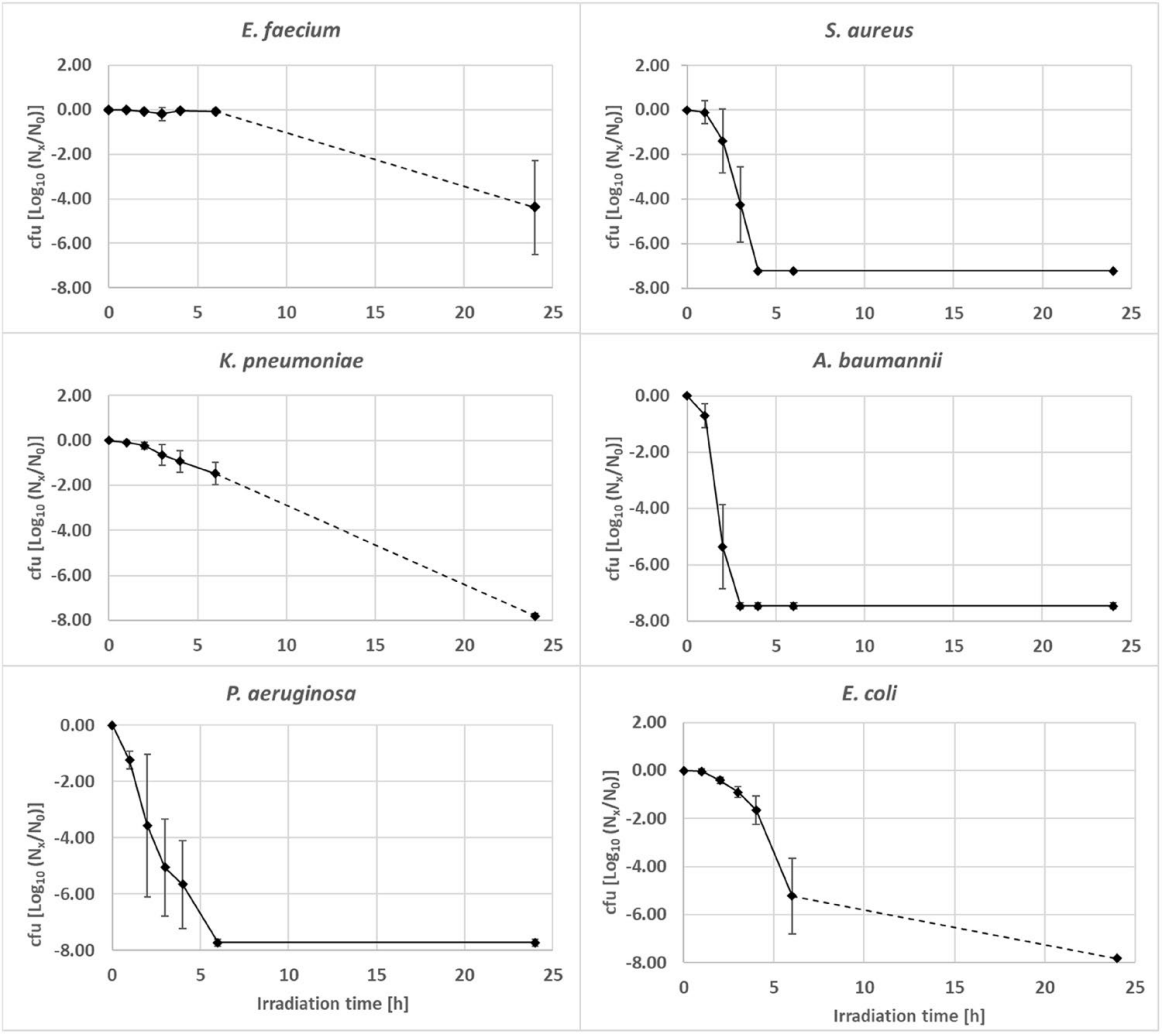
Log_10_-reduction rate of facultative pathogenic bacteria after different hours of irradiation (254 W/m^2^) with broad range bue light (380–500 nm). The means of three independent experiments are shown for each reference bacterium. The standard deviations are indicated by whiskers. Broken lines represent exposure times without any distinct cultivation experiments assuming values above total reduction rate. Solid lines represent the continous measured reduction rates of the different reference bacteria reaching the point of total reduction before the long term aBL irradiation (24 h)

The selected reference strains of the ESKAPE-group were irradiated with broad spectrum aBL (245 W/m^2^; Fig. 3), and the antibacterial efficiency was investigated by determination of bacterial concentrations (cfu/mL). Figure 3 describes the different inactivation dynamics of reference bacteria exposed to aBL treatment in a time-dependent manner. Since the focus of this study is on wastewater treatment with aBL, bacterial suspensions were used that were comparable to the 16S rDNA qPCR results from real slaughterhouse wastewater (Figs. 1 and 2). In addition, increased cell numbers were necessary to evaluate the log_10_ reduction potential of the applied aBL irradiation. Taxon-specific differences became visible, when high-density bacterial suspensions were used. It became obvious that after 6 h of irradiation, the impact of aBL on the bacterial reference strains offers various sensitivities. Whereas cfu values of *E. faecium and K. pneumoniae* persisted at a high level similar or close to initial untreated sample (0 h), all other strains (i.e., *E. coli*, *S. aureus*, *P. aeruginosa*, and *A. baumannii*) were much stronger affected by the blue light. Here, the log_10_ units reduction ranged up to 8 log_10_ units in maximum (Fig. 3). The reference strains of *A. baumannii*, *P. aeruginosa*, and *S. aureus* showed a reduction of > 7 log_10_ units within the first 6 h of irradiation. In addition, the reduction for *E. coli* was about more than 5 log_10_ units. Similar to *K. pneumoniae*, *E. faecium* was less sensitive to blue light and no distinct reduction by aBL became visible. In consequence, it is possible to categorize the used different bacterial reference strains of the ESKAPE-group in 1) highly sensitive with *S. aureus*, *A. baumannii*, *and P. aeruginosa*; 2) sensitive with *E. coli* as a representative bacterium of the large group of *Enterobacteriacae*; and 3) resistant or less sensitive against aBL with *E. faecium* and *K. pneumoniae.*

**Fig. 4:**
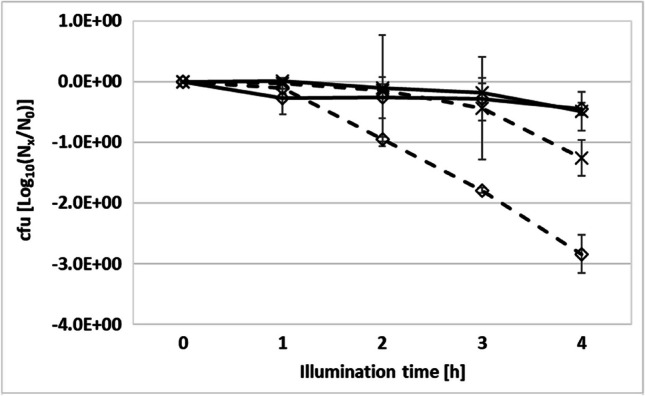
Effects of various culture growth media and growth phases of the aBL insensitive *K. pneumoniae* reference strain. High-nutrient LB medium (diamond) and mineral medium BM2 (cross) were used. Different cultivation experiments were evaluated using overnight cultures representing stationary growth phase (solid lines), as well as cultured from freshly prepared exponentially growing bacteria culture (broken lines). Colony forming unit determination were run from 0 h (initial control) to 4 h of aBL irradiation (542 W/m^2^). Results are means of three independant experiments and standard deviations are given as whiskers

To test the possible dependence of inactivation by aBL on growth phases and nutrient availability, the aBL insensitive reference bacterium *K. pneumoniae* was grown in a high nutrient medium (LB, Luria-Broth) and in a mineral medium (BM2, basal-medium 2) with glucose as single carbon-hydrate source. Overnight cultures (stationary growth phase) and bacteria from the exponential growth phase in high- and low-nutrient growth media were used for aBL irradiation experiments (543 W/m^2^; 30 °C) (Fig. 4). The late stationary phase cultures showed a less sensitivity to aBL during 4 h of irradiation in contrast to cultures harvested from the exponential growth phase. In both experimental approaches, the starting cell count was analyzed with about 8.0 log_10_ units cfu/mL. Here, the aBL impact on the *K. pneumoniae* suspensions was comparable in case of high- and low-nutrient cultivation conditions. The cell numbers were found to be about 1.0 × 10^7^ cfu/mL in both culture experiments. The maximal reduction capacityies were found with a maximum of only 1 log_10_ units after 4 h illumination.

Reduction values gained from the exponentially growing bacteria offered slightly more antibacterial impacts especially when cultivated in high-nutrient LB medium with about 3 log_10_ units which corresponded to about 1.0 × 10^5^ cfu/mL. The antibacterial impact of blue light on exponentially growing cultures in low-nutrient BM2 medium was much lower with about 1 log_10_ unit after 4 h of irradiation. Finally, it could be shown that the different growth phases and the better nutrient supply have an influence on the inactivation by aBL in *K. pneumoniae*, which might result from an increased metabolic activity.

A comprehensive elimination of the bacteria from the ESKAPE-group should be the goal of inactivation in various water matrices. To achieve this, it is necessary to integrate innovative approaches that optimize aBL treatment in order to inactivate aBL-insensitive bacteria.

**Fig. 5:**
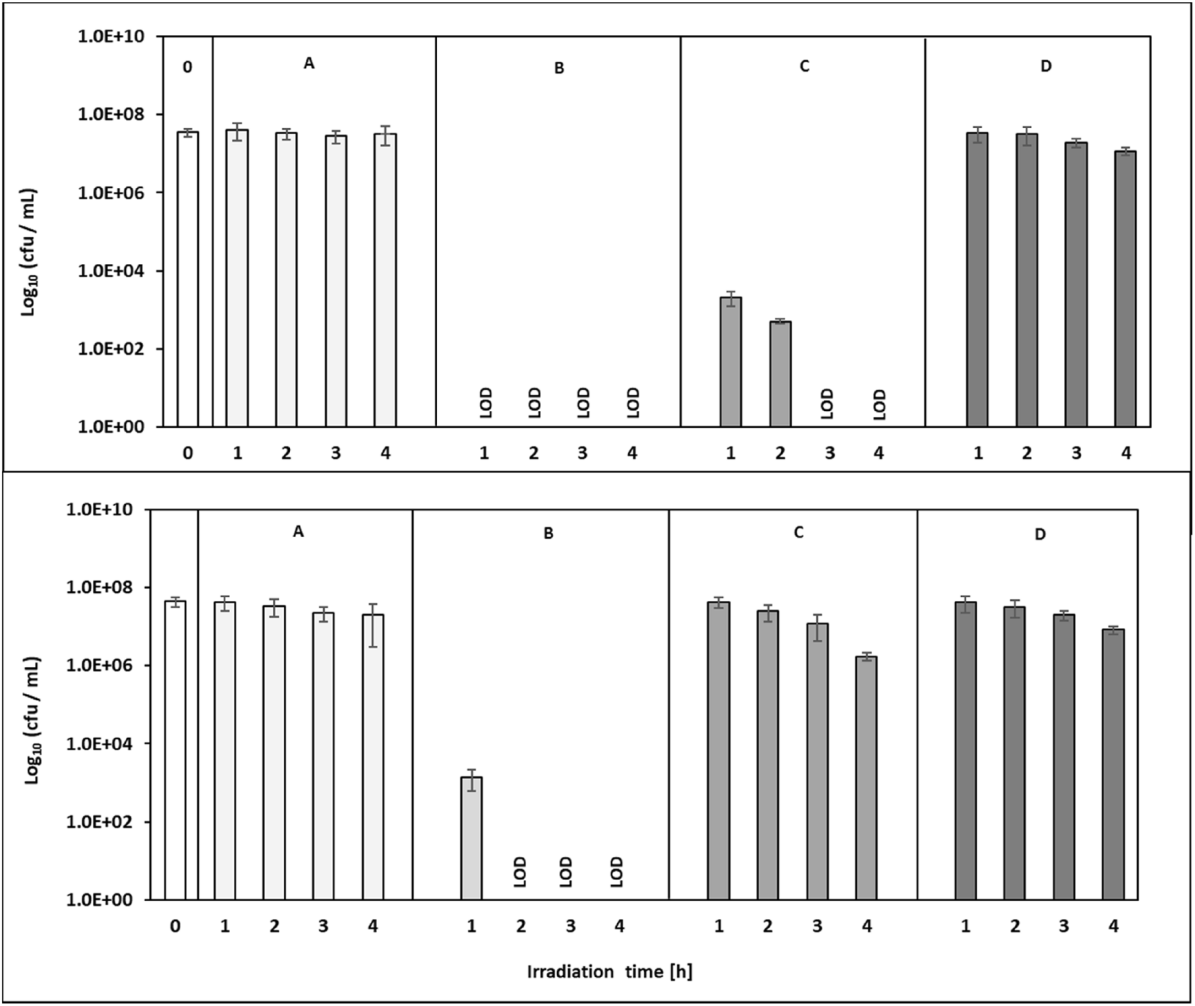
Bacterial suspension of aBL robust *K. pneumoniae* (top) and *E. faecium* (bottom) (both early stationary growth phase cultures for 1 to 4 h irradiation): (**0**) initial sample, (**A**) aBL without TMPyP, (**B**) aBL with TMPyP (1.0x10^-6^ M); (**C**) aBL with TMPyP 1.0 × 10^−7^ M, and (**D**) aBL plus TMPyP 1.0 × 10^−8^ M, respectively. The aBL irradiation was performed using 543 W/m^2^. The data are the means of different dilutions steps calculated to cfu/mL. LOD, below the detection limit. Standard deviation are given in whiskers

Both insensitive reference bacteria *E. faecium* and *K. pneumoniae* were selected for photo-sensitizer experiments enhancing antibacterial efficiency of aBL. Here, strong additional effects of aBL treatment were detected already from 1 h of irradiation in the presence of the photo-sensitizer concentration of 1.0 × 10^−06^ M solution (approx. 1.35 mg/L) (Fig. 5). Control experiments in dark showed the non-bacterial toxicity of the TMPyP photo-sensitizer (data not shown).

During an irradiation time of 4 h, the control suspensions without TMPyP confirmed the insensitivity of both *K. pneumoniae* and *E. faecium,* which showed no decrease of cfu per mL over time (Fig. 5A). But, a 1.0 × 10^−6^ M addition of TMPyP to the bacterial suspensions showed strong reduction impacts almost after 1 h of aBL treatment. For *K. pneumoniae* a decreased colony counts with > 4 log_10_ units was measured, whereas no colonies were detectable for *E. faecium* (Fig. 5B). In case of longer incubation times > 1 h working with 1.0 × 10^−6^ M TMPyP and aBL no cfu was measured indicating the elimination of these both aBL insensitive bacterial strains (Fig. 5B).

The application of 1.0 × 10^−7^ M or 1.0 × 10^−8^ M TMPyP approaches together with aBL showed weaker reduction capacities for both reference bacteria *K. pneumoniae* and *E. faecium* (Fig. 5C and D).

### Combinatory inactivation strategy with raw sewage from slaughterhouses

**Fig. 6:**
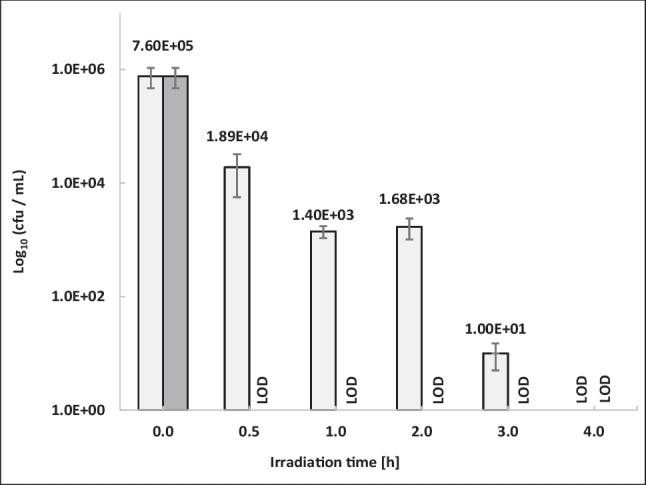
Native slaughterhouse raw sewage treated with aBL (543 W/m^2^) in addition with 1.0 × 10^−6^ M (dark bar) or 1.0 × 10^−7^ M TMPyP (light bar) for 0.5, 1.0, 2.0, 3.0, and 4.0 h. The initial cell count (0.0 h) cultivated on LB medium derived from the same sewage sample. LOD, below the detection limit. Standard deviations are given in whiskers

The application of this combinatorial inactivation strategy against entire bacterial populations was tested on a real raw sewage from a poultry slaughterhouse (Fig. 6). Firstly, the impact of aBL on the total population of raw sewage was tested by preparing 1:10 dilutions of raw sewage due to the high turbidity of the samples. After 4 h, it was found that the aBL irradiation without TMPyP treatment of the total population was resulted in a weak reduction of about 1 log_10_ unit (data not shown). But, the presence of the photo-sensitizer TMPyP (1.0 × 10^−7^ M) together with an aBL irradiations could decrease the bacterial concentration of the total population of diluted raw sewage below LOD during 4 h of treatment. More successfully, the photo-sensitizer concentration of 1.0 × 10^−6^ M TMPyP reduced the colony counts below LOD already after 0.5 h of treatment (Fig. 6). Again, combinatorial approaches reducing unwanted bacterial contaminations were shown to be highly effective. However, wastewater parameters such as turbidity due to organic or abiotic loads must be taken into accounts. Therefore, in the data shown with raw wastewater, only diluted approaches could be used. However, it must also be emphasized that these are small-volume laboratory experiments in a static approach. It remains to be seen to what extent these combinatory strategies can be applied in modified up-scaled flow reactors.

## Discussion

Wastewater from poultry and pig slaughterhouses is a hotspot for antibiotic resistances and facultative pathogenic bacteria. Nowadays, the slaughterhouse technologies used have been adapted to the new circumstances, where so-called slaughter lines are now used for one animal species and can achieve enormous throughputs. For pigs, the slaughter capacity can reach over 20,000 animals per day; for chickens, the largest plants slaughter several hundred thousand animals per day. Due to food safety and hygiene requirements, savings in energy and water consumption are limited to a certain degree. The wastewater from slaughterhouses is organically highly contaminated and demonstrates high chemical oxygen demand (COD) values. Therefore, such industries usually have wastewater pre-treatment at their plant before conditioned wastewaters are released to municipal sewer systems. This was also the case of our studies, where different wastewaters were analyzed for facultative pathogenic bacteria and thus also ARGs emitted from large slaughterhouses.

The concentrations of six different facultative pathogenic bacteria based on the WHO priority list of the ESKAPE group (WHO 2019a), which are also known to be carriers of clinically relevant resistances, were quantified by molecular biology approaches in the effluent of poultry and pig slaughterhouses that carry out large-scale slaughtering. In addition, a total of 14 antibiotic resistance genes of different priorities for human medicine were selected, whose concentrations were also quantified. Only living, intact facultative pathogenic bacteria or ARGs from living bacteria were quantified by qPCR in order to show reduction efficiencies of existing and innovative technologies. In comparison with other studies, who identified hotspots of antibiotic resistance dissemination (e.g., Alexander et al. 2022; Paulus et al. 2019; Voigt et al. 2020), we have also demonstrated that raw sewage from poultry and pig slaughterhouses is highly contaminated not only with facultative pathogenic bacteria but also with various categories of clinically relevant ARGs, including ARGs against the group of reserve antibiotics. Conventional biological or advanced (oxidative) treatments significantly decreased the concentration of the different gene targets by several log_10_ units depending on the bacterial target, after but were not eliminated. Therefore, the application of treatment procedures for a more effective reduction of hygienically relevant bacteria and clinically important ARGs, aiming at a stronger reduction/elimination of these microbiological parameters, is recommended.

The overall aim should be to minimize the spread of ARB and ARGs from hotspots disseminated into the public wastewater network and subsequent into the environment. Targeted and innovative light-based on-site measures relieve downstream wastewater areas of AMR contamination, reducing their release into the environment (Mulani et al. 2019). On the one hand, this concerns municipal central wastewater treatment plants, but on the other hand also receiving waters that take in the treated wastewater directly. Especially in times of climate change with rising temperatures and dry periods, the percentage of treated wastewater in receiving waters can increase. This is associated with an increase in the concentrations of AMB and ARGs in these surface waters. Increased water temperature can then also enhance the persistence of these bacteria in the aquatic environment. The risk of contamination for humans is particularly present when protected assets in the type of water usages are affected. These are, for example, bathing waters, recreational areas, drinking water reservoirs, and agricultural areas that are irrigated with re-used waters. But increasingly frequent heavy rainfall events also lead to run-off of ARB, especially from agricultural land fertilized with manure from livestock farms. Hembach et al. (2022) already showed the contamination of liquid manure with ARGs.

As a proof of principle investigation, we demonstrated with the help of a laboratory pilot system the destruction of ARB/ARGs in contaminated wastewater from slaughterhouse with a blue light–based technology in combination with the photo-sensitizer TMPyP porphyrin molecule. It was discussed that different wavelengths are responsible for activation of diverse photo-sensitive molecules involved in bactericidal activities (Hessling et al. 2017; Hoenes et al. 2019). Whereas short blue light of 405 nm is known to activate porphyrins, longer wavelengths up to 450 nm have a stronger interaction with endogenous flavins as photo-sensitizers (Hoenes et al. 2019; Plavskii et al. 2018). However, it also must be said that it is currently not clear how different bacterial genera react to visible light of the same wavelength (Tomb et al. 2018).

In connection with aBL irradiation, a comparison with the widely used ultraviolet (UV) light, as an applied photo-based antimicrobial technology, is appropriate in order to look at the advantages and disadvantages of both methods. Especially UV-C (100–289 nm) mainly has gemicidal effects. These effects are caused by the DNA and RNA as well as proteins absorbing irradiation at the respective wavelength. The absorption curve of DNA reaches its maximum at a wavelength of 260 nm and its local absorption minimum close to 280 nm. As a consequence, the germicidal effect for microbes is achieved at a wavelength of 260 nm (Süß et al. 2009; Jungfer et al. 2007). The effectiveness of UV light in the biological inactivation primarily results from the fact that DNA molecules absorb UV photons with peak absorption at 265 nm. In case of lethal damages, the DNA replication is blocked due to DNA alterations, mainly thymine dimers, which ultimately result in reproductive cell death. During evolution, bacteria generally possess molecular mechanisms, such as photo-reactivation and dark repair systems, to restore such DNA lesions. All mechanisms are regulated by the expression of the *rec*A gene, the key gene in this system (Sinha and Hader 2002; Jungfer et al. 2007).

In contrast to UV inactivation, the aBL photo-based irradiation is activating the reactive oxygen molecules (ROS) in the bacterial cells. These reactive oxygen molecules are targeting different cellular structures inducing lethal impacts on the microorganisms. Hence, in case of UV irradiation the nucleic acid alterations are the main underlying mechanism, where DNA-directed repair mechanisms were developed in bacteria during evolution. In difference, up to now no repair mechanisms are known to repair the manifold target structures impaired by aBL irradiation (Hadi et al. 2020). Hence, a more sustainable application of aBL in combination with porphyrin photo-sensitizer is hypothesized. In principle, the use of LED-based photo-reactors is significantly more cost saving than conventional lamp designs such as those used for UV-C irradiation. The UV medium-pressure radiation source with 1000 W power input generates a radiation flux of approx. 21.2 W in the range of 405 nm. A commercially available LED generates a radiation flux of 46 W at a power input of 100 W. If we increase this value by a factor of 10 to a LED spotlight with a power input of 1000 W, we would arrive at 460 W. Roughly speaking, an LED radiation source is 21.7 times more efficient in the 405 nm range than a classic medium-pressure radiation source with the same energy input and with the advantage that no undesired side reactions occur (personal communication with light industry company).

However, a direct comparison of the presented LED-based blue light system would only make sense once an up-scaling for a flow reactor with appropriately adapted LED light sources has been tested. Here, the energy inputs for the inactivation of critical bacteria (see SI Table 4) refer exclusively to the stagnation reactor used for small-volume laboratory experiments.

The recent studies have shown that facultative pathogenic bacteria of the ESKAPE group demonstrated a diverse susceptibility for the broad range of aBL (405–450 nm wavelength). This phenomenon was independent from the Gram properties of the reference bacteria. *E. faecium* and *K. pneumoniae* demonstrated a strong insensitivity against aBL compared to the other reference bacteria. But, the application of the porphyrin TMPyP enhanced the inactivation capacity of aBL in a similar range like the aBL sensitive strains. This enhancement is aBL dependent, since control experiments demonstrated no inactivation of the reference bacteria without previous blue light irradiation (dark exposure) as well as the non-toxicity of the photo-sensitizer molecule TMPyP (data not shown). It has to be mentioned that aBL impact might also be influenced by particular components and ingredients of the raw sewage increasing the turbidity and transparency of the water matrix. Actually, due to the high turbidity we needed to dilute the raw wastewater samples released from slaughterhouses for aBL irradiation. No transmission of the blue light was possible in case of undiluted samples. In that concern, a previous filtration step could benefit the reduction performance for facultative pathogenic bacteria and ARGs, especially in case raw wastewaters. Due to the fact that we have initially used a stagnation approach with a low sample volume, the inactivation capacities of up-scaled flow aBL reactors have to be analyzed, designed for a modular implementation in sewer systems.

Actually, aBL treatment as a single inactivation approach demonstrated an apparently insufficient reduction capacity in real slaughterhouse sewage systems. Hence, aBL treatment was linked with the photo-sensitizer TMPyP application. Similar to the previous experiment with reference bacteria of the ESKAPE-group, the TMPyP photo-sensitizer addition increased the reduction using native slaughterhouse wastewater in a concentration-dependent manner. At present, it is still unclear why the effect of the photo-sensitizer TMPyP is restricted to such a limited concentration range when working with these aBL-insensitive reference bacteria *K. pneumoniae* or *E. faecium*, and native wastewater samples. In principle, the results showed that it is possible to combine antimicrobial strategies to inactivate a potentially wide range of hygienically critical bacteria including insensitive bacteria.

It is hypothesized that the application of a specific concentration of TMPyP increases the target molecule amount responsible for the generation of reactive oxygen substances being the causative agents for bacterial killing rate impacts. Furthermore, threshold values of endogenous porphyrin amounts are a critical issue, since most effective concentrations of photo-sensitizer TMPyP were detected to be 10^−6^ M.

However, it should be noted that not the harmless, native water flora is of hygienic relevance, but facultative pathogenic bacteria with ARGs are of clinical relevance and should be inactivated. The inactivation effect on wastewater-borne active viruses or protozoa with hygienic relevance is also still unclear. These microbes are not considered in this study. Finally, these findings demonstrate a promising starting point of an alternative strategy to effectively eliminate a broad spectrum of (pathogenic) bacteria from wastewater by using aBL, especially in combination with additional inactivation approaches. As a near future perspective, modular designs of this aBL-based technology should allow installation at hazardous critical hotspots in process lines.

## Conclusion

Poultry and pig slaughterhouses produce wastewater with high concentrations of clinically relevant bacteria of the ESKAPE group and ARGs. Different clusters of critical ARGs are detectable in slaughterhouse raw wastewater and show different reduction behaviors by conventional treatment processes. On-site treatments by biological wastewater treatment and ozonation showed certain reduction capacities, which were not effective enough for all investigated ARGs and facultative pathogenic bacteria. In this respect, it has not yet been possible by national and international authorities to define threshold values at which specific hygienically treatment of wastewaters should be implemented. In general, the occurrence of resistances to reserve antibiotics is described as a particularly critical issue and its dissemination should be avoided. Hence, The application of aBL LED irradiation demonstrated differences in the killing efficiency across the selected representatives of the ESKAPE group microorganisms. The reference organisms could be categorized to “highly sensitive,” “sensitive,” and “insensitive” for aBL inactivation. aBL in interaction with porphrin photo-sensitizer TMPyP (10^−6^ M) allowed a reduction/elimination of reference bacteria and also bacteria in native slaughterhouse wastewater. Hence, combinatorial methods are more successful in protecting downstream areas of the aquatic use pathway.

### Supplementary information

ESM 1

## Data Availability

Data are available from the corresponding author upon reasonable request.
