# Supplementary material for: Antibiotic resistances from slaughterhouse effluents and enhanced antimicrobial blue light technology for wastewater decontamionation
Source: Environ Sci Pollut Res Int. 2023 Sep 29;30(50):109315–30. doi: 10.1007/s11356-023-29972-x (PMC10622382; doi:10.1007/s11356-023-29972-x)
Supplement: Supplementary file 1 — ESM 1 [file 11356_2023_29972_MOESM1_ESM.docx]

**Supporting Information**

Antibiotic resistances from slaughterhouse effluents and enhanced antimicrobial blue light technology for wastewater decontamionation.

Cong Xiaoyu^1^, Krolla Peter^1^, Khan Umer Zeb^1^, Savin-Hoffmeyer Michael^2^, Schwartz Thomas^1^*

^1^Karlsruhe Institute of Technology (KIT), Institute of Functional Interfaces (IFG), Hermann von Helmholtz Platz 1, 76344 Eggenstein-Leopoldshafen, Germany.

^2^University Clinic of Bonn, Institute of Hygiene and Public Health (IHPH), Venusberg-Campus 1, 53127 Bonn, Germany

SI Table 1: Primer sequences, calibaration line equation, efficiency values, detection limits, and correlation coefficientvalue oft he calibration lines, amplicon sizes, references. The table clustered he qPCR systems used for the quantification of facultative pathogenic bacteria and antibiotic resistence genes according to Hembach et al. (2017, 2022) and Alexander et al. (2020; 2022)

| Target | Primer sequence | Equation of the calibration curve | Amplicon size | efficiency | R² | LOD | Control strain | reference |
| --- | --- | --- | --- | --- | --- | --- | --- | --- |
| **Facultative pathogenic bacteria** | | | | | | | | |
| *Enterococcus* spp. | Fwd: AGAAATTCCAAACGAACTTG  Rev: CAGTGCTCTACCTCCATCATT | F(x)= ‑3,585x+35,283 | 93 bp | 90,1 % | 1,000 | 65 | *E. faecium*  DSM20477 | (Frahm und Obst 2003) |
| *E. faecalis* | Fwd: CACCTGAAGAAACAGGC  Rev: ATGGCTACTTCAATTTCACG | F(x)= -3,472x+35,447 | 475 bp | 94,1 % | 0,999 | 6 | *E. faecalis*  ATCC51299 | (Depardieu et al. 2004) |
| *P. aeruginosa* | Fwd: AGCGTTCGTCCTGCACAAGT  Rev: TCCACCATGCTCAGGGAGAT | F(x)= ‑3,282x+35,276 | 81 bp | 101,7 % | 0,999 | 3 | *P. aeruginosa*  DSM1117 | (Clifford et al. 2012) |
| *K. pneumoniae* | Fwd: ACGGCCGAATATGACGAATTC  Rev: AGAGTGATCTGCTCATGAA | F(x)= ‑3,387x+38,844 | 68 bp | 97,4 % | 0,998 | 18 | *K. pneumoniae*  DSM30104 | (Clifford et al. 2012) |
| *A. baumannii* | Fwd: GTTGTGGCTTTAGGTTTATTATACG  Rev: AAGTTACTCGACGCAATTCG | F(x)= ‑3,380x+35,679 | 94 bp | 97,6 % | 1,000 | 31 | *A. baumannii*  DSM30007 | (Clifford et al. 2012) |
| *E. coli* | Fwd: GCATCGTGACCACCTTGA  Rev: CAGCGTGGTGGCAAAA | F(x)= ‑3,361x+35,797 | 59 bp | 98,4 % | 0,994 | 4 | *E. coli*  DSM1103 | (Clifford et al. 2012) |
| **Antibiotic resistance genes** | | | | | | | | |
| *erm*B | Fwd: TGAATCGAGACTTGAGTGTGCAA  Rev: GGATTCTACAAGCGTACCTT | F(x)=‑3,328x+35,901 | 71 bp | 100 % | 1,000 | 16 | *S. hyointestinalis*  DSM20770 | (Alexander et al. 2015) |
| *Int*I1 | Fwd: GCCTTGATGTTACCCGAGAG  Rev: GATCGGTCGAATGCGTGT | F(x)= ‑3,472x+34,720 | 196 bp | 94,1 % | 1,000 | 126 | *E. coli* pNORM | (Rocha et al. 2018) |
| *tet*M | Fwd:GGTTTCTCTTGGATACTTAAATCAATC  Rev: CCAACCATAAATCCTTGTTCRC | F(x)=‑3,424x+38,747 | 88 bp | 95,9 % | 0,998 | 4 | *E. coli* DH5α | (Peak et al. 2007) |
| *bla*_TEM_ | Fwd: TTCCTGTTTTTGCTCACCCAG  Rev: CTCAAGGATCTTACCGCTGTTG | F(x)=‑3,303x+38,559 | 112 bp | 100,8 % | 0,999 | 80 | *E. coli* pNORM | (Rocha et al. 2018) |
| *sul*1 | Fwd: CGCACCGGAAACATCGCTGCAC  Rev: TGAAGTTCCGCCGCAAGGCTCG | F(x) ‑3,387x+39,802 | 161 bp | 97,6 % | 0,999 | 80 | *E. coli* pNORM | (Rocha et al. 2018) |
| *bla*_CTX‑M_ | Fwd: CGCTTTGCGATGTGCAG  Rev: ACCGCGATATCGTTGGT | F(x)= ‑3,504x+34,255 | 551 bp | 92,9 % | 1,000 | 93 | *E. coli* pNORM | ( Rocha et al. 2018) |
| *bla*_CTX‑M‑32_ | Fwd: CGTCACGCTGTTGTTAGGAA  Rev: CGCTCATCAGCACGATAAAG | F(x)= ‑3,517x+37,800 | 155 bp | 92,5 % | 1,000 | 235 | *E. coli* pNORM | (Rocha et al. 2018) |
| *bla*_OXA‑48_ | Fwd: TGTTTTTGGTGGCATCGAT  Rev: GTAAMRATGCTTGGTTCGC | F(x)= ‑3,540x+36,913 | 177 bp | 91,6 % | 0,998 | 92 | *K. pneumoniae*  TGH Isolate 2 | (Monteiro et al. 2012) |
| *van*A | Fwd: TCTGCAATAGAGATAGCCGC  Rev: GGAGTAGCTATCCCAGCATT | F(x)= ‑3,541x+33,078 | 376 bp | 91,6 % | 1,000 | 43 | *E. faecium* B7641 vanA | (Klein et al. 1998) |
| *mec*A | Fwd: CGCAACGTTCAATTTAATTTTGTTAA  Rev: TGGTCTTTCTGCATTCCTGGA | F(x)=‑3,327x+34,887 | 91 bp | 99,8 % | 1,000 | 11 | *S. Aureus* A1 | (Volkmann et al. 2004) |
| *mcr*‑1 | Fwd: GGGCCTGCGTATTTTAAGCG  Rev: CATAGGCATTGCTGTGCGTC | F(x)=‑3,386x+35,349 | 183 bp | 97,4 % | 0,999 | 8 | *E. coli* NRZ‑14408 | (Hembach et al. 2017) |
| *bla*_NDM_ | Fwd: TTGGCCTTGCTGTCCTTG  Rev: ACACCAGTGACAATATCACCG | F(x)= ‑3,293x+35,877 | 82 bp | 101,2 % | 0,999 | 66 | *K. pneumoniae*  ATCC BAA‑2146 | (Monteiro et al. 2012) |

SI Table 2: Water sample getting from poultry slaughterhouse. Abundance of facultative pathogenic bacteria (A), abundance of “commonly occurring resistance genes” (B), abundance of “intermediately occurring resistance genes” (C), and abundance of “rarely occurring resistance genes” (D). The data from each sampling campaign are listed together with the standard deviations SD.

A

B

C

D)

SI Table 3: Water sample getting from pig slaughterhouse. Abundance of facultative pathogenic bacteria (A), abundance of “commonly occurring resistance genes” (B), abundance of “intermediately occurring resistance genes” (C), and abundance of “rarely occurring resistance genes” (D). The data from each sampling campaign are listed together with the standard deviations SD.

A

B

C

D

**SI Table 4:** Radiant intensity data were collected with a calibrated spectrophotometer (FLAME-S-XR1-ES, with optical fibre QP400-2-SR-BX; OceanInsight, Ostfildern, Germany) from the LED light source inside the incubator. Due to the fixed light emission of the LEDs, different light intensities, high and low, were performed by changing the light source – reaction vial distance.

| **Irradiation** | **High intensity** | **Low intensity** |
| --- | --- | --- |
| **Radiant intensity [W/cm²] corresponds with J/s*cm^2^** | **8,33E-02** | **5,43E-02** |
| **Irradiation time [h]** | **Time dependent energy input [J/cm²]** | |
| **1** | **300** | **196** |
| **2** | **600** | **392** |
| **3** | **900** | **588** |
| **4** | **1200** | **784** |

**Figure S1**:

**
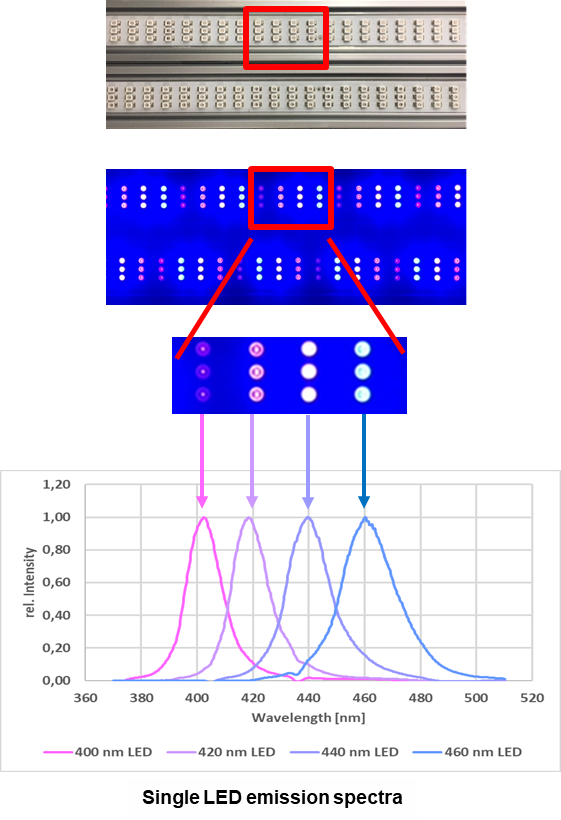
**

Figure S1: Exemplary: normalized entire emission spectrum of the LED bar (top); normalized emission spectra of each LED type on LED bar (bottom). A qualtitative characterization of the emitted spectrum was done with a spectrometer (FLAME-S-XR1-ES, OceanInsight, Ostfildern, Germany).
